# Gender difference in suicidal ideation and related factors among rural elderly: a cross-sectional study in Shandong, China

**DOI:** 10.1186/s12991-019-0256-0

**Published:** 2020-01-14

**Authors:** Lu Lu, Lingzhong Xu, Xiaorong Luan, Long Sun, Jiajia Li, Wenzhe Qin, Jiao Zhang, Xiang Jing, Yali Wang, Yu Xia, Yaozu Li, An’an Jiao

**Affiliations:** 1 School of Public Health, Shandong University, Jinan, 250012 China; 2 NHC, Key Laboratory of Health Economics and Policy Research, Shandong University, Jinan, 250012 China; 3 Center for Health Economics Experiment and Public Policy Research, Shandong University, Jinan, 250012 China; 4 Department of Nursing Management of Qilu Hospital, Shandong University, Jinan, 250012 China

**Keywords:** Suicidal ideation, Gender difference, Rural elderly, China

## Abstract

**Background:**

Suicide is a global public health problem which has significant negative influence on individuals, families and the society. The aim of this study is to investigate the prevalence of suicidal ideation and related factors among elderly people in rural China, and further examine the gender differences of suicidal ideation.

**Methods:**

Data were collected from the 2017 Survey of the Shandong Elderly Family Health Service, which was conducted by Shandong University. A total of 5514 elderly aged 60 and above from rural Shandong were included in this study. Binary logistic regression model was performed to examine the gender difference towards suicidal ideation, and to identify the influencing factors of suicidal ideation by gender among elderly.

**Results:**

7.7% rural elderly reported suicidal ideation in the past 12 months in Shandong, China. The prevalence of suicidal ideation among females was significantly higher than that among males (*P* < 0.001). Education level, debts, stress of daily life, loneliness and psychological distress were significantly related to suicidal ideation in both males and females. Besides, negative life events and life satisfaction were found to be significantly associated with suicidal ideation in females but not in males.

**Conclusions:**

There was a significant gender difference towards suicidal ideation among rural elderly in Shandong, China. So, gender difference should be considered when medical practitioners and public health workers seek to prevent and manage suicidal ideation among rural elderly, which will be important to develop strategies for coping with risk factors for suicidal ideation among males and females. In other words, more attention should be paid to females who had encountered negative life event or with lower life satisfaction.

## Background

Suicide is a serious public health issue in the worldwide, which causes a heavy economic, social and psychological burden on the individuals, families and society. According to the World Health Organization (WHO), suicide accounted for 1.4% of all deaths worldwide, making it the 18th leading cause of death in 2016 [1]. Globally, there were an estimated 793,000 suicide deaths in 2016, which indicated an annual global age-standardized suicide rate of 10.5 per 100,000 population [2]. The WHO suicide prevention report pointed out that the prevalence of suicide in China was 7.8 per 100,000 people in 2012 [3] and ranked as the fifth leading cause of death among the general population and contributed to 42% of all suicide worldwide and 56% of the world’s female suicide [4]. With the aging of population, the prevalence of suicide among elderly is increasingly higher, which becomes one of the most important public health issues we should of concern. A report from WHO showed that suicide rate for older adults is highest for those aged 70 years or over in almost all regions of the world [3]. Some previous studies also indicated that the elderly has the highest suicide rates [5, 6]. Another study showed that seniors aged 65 years and over had the highest rate of completed suicide in China, which reached 44.3–200 per 100,00 [7]. It is worth noting that there are some special characteristics of suicide rates in China compared to western countries, such as the suicide rate among the rural elderly is much higher than that among the urban elderly [7, 8], and the rate among females is higher than that among males [4, 9]. China is one of few countries with higher female suicide rates in the worldwide, but little is to known about the gender difference in suicidal ideation among Chinese rural elderly. Therefore, suicidal ideation among Chinese rural elderly and the difference between males and females deserve more attention.

Suicidal ideation is the early process of suicide and it always presents before suicide completion. According to available evidence, suicidal ideation can be regarded as a trigger for attempted and completed suicide. Thus, understanding the potential risk factor of suicidal ideation in the initial stages of suicide is important in the suicide prevention. The prevalence of suicidal ideation in seniors in China ranges from 2.2 to 21.5%, and suicidal ideation increases gradually with age in older adults [10]. Many factors have been found to be associated with suicidal ideation among older adults, including age [11, 12], gender and marital status [13], education level [14, 15], living arrangement [16], economic status [17], social support and depression [18], which can be classified into three groups: sociodemographic factors, socio-environmental factors and psychological factors. Besides, factors related to physical health such as chronic disease and disability [19, 20] were also risk factors for suicidal ideation.

Among these influencing factors, gender is one of the most significant factors which we should consider for suicidal ideation, given that gender difference in the suicide [21] and suicide attempts [22, 23] have been confirmed. Gender difference towards suicidal ideation reflects the difference not only in demographic characteristics, but also in socioeconomic circumstances and health conditions. A previous study conducted in South Korea revealed that the prevalence of suicidal ideation was higher among female adolescents than among their male counterparts [24]. Another study conducted in Malaysia showed that suicidal ideation was higher among males compared with females [25]. Similarly, a study from China mainland showed that girls were significantly more likely than boys to report both suicidal ideation and attempts [26]. Another study from China Taiwan showed that girls who reported quarrels with parents had the highest level of suicidal ideation before age 18 [27]. However, studies mentioned above mainly focused on adolescent students or young adults for gender difference in suicidal ideation. To our knowledge, only a few studies focused on older adults and there was a lack of research investigated the difference towards suicidal ideation by gender among elderly in rural China. Thus, our study aims to examine the gender difference in suicidal ideation and related factors among rural elderly in Shandong, China.

## Methods

### Data and sample

Our data were collected from the 2017 Survey of the Shandong Elderly Family Health Service, which was conducted by Shandong University. Multi-stage random sampling was applied to select participants: in the first stage, 6 counties were selected from 137 counties as the primary sampling units (PSUs) throughout the eastern, central and western regions of Shandong Province (which were divided into 3 districts and 3 counties that represented urban and rural areas separately). From each PSU, 18 villages in rural area and 18 communities in suburban and urban area were selected as the secondary sampling units (SSUs). In the third stage, based on the roster of the residents by age and the total elderly population of each selected site provided by the local residential committee, an average of 66 individuals were stratified and randomly selected from each SSU making up the total sample. The eligible participants for this survey were those aged 60 or older with local household registrations at the time of the interview. In total, 7070 individuals were included in the final sample. Our study focused on respondents in the rural area with a sample of N = 5514.

### Dependent variable

In our study, suicidal ideation was assessed by using a question of “Have you ever seriously thought about committing suicide in the past 12 months?” from the US National Comorbidity Survey [28]. If the answer was “yes”, suicidal ideation was coded as “1”. If the answer was “no”, suicidal ideation was coded as “0”.

### Independent variable

The social demographic characteristics included gender, age, marital status, education level, self-rated economic status and debt status. Gender was categorized as male and female. The age of the participants was grouped into three types: 60–69, 70–79 and 80~years. Marital status was categorized into married and others (include unmarried, divorced and widowed). Education level was classified into three categories: illiterate, primary school and middle school or above. Self-rated economic status was measured by asking participants to rate their economic status with four options: wealthy and not worried about livelihood, not wealthy but not worried about livelihood, not wealthy and worried about livelihood, poor and worried about livelihood. Response of “wealthy and not worried about livelihood” was identified into the “good” category, and other responses were recoded as “moderate or bad”. Debt status was measured by a question of “Do your family have any debts at present?”. The answer included “yes” and “no”.

Family-related factors included living arrangement, relationship with family, stress of daily life, and negative life events. Living arrangement was divided into “living alone” and “living with others”. We asked participants with a question of “How do you feel about the relationship with your family?” and the answer was categorized into “good” and “moderate or bad”. Stress of daily life was assessed by asking participants rate their life stress with five options: very high, high, moderate, low, none. We recorded the former four responses as “yes” and the last one as “no”. Negative life events were assessed by asking a question of “Have you encountered any major setbacks or unexpected misfortunes in your life and work in the past two years, such as natural hazards, diseases, accidents, death of a family member, lawsuits, dispute, etc.?” The response options included “yes” and “no”.

Physical health status included self-rated health, non-communicable chronic diseases (NCDs) condition, and activities of daily living (ADL) status. Self-rated health status was assessed by asking the participants rate their health condition with a single question: “would you say that your health has on the whole been excellent, good, moderate, bad, or very bad in a month?” Response of “excellent” and “good” was classified into the “good” category, and remaining were recoded as “moderate or bad”. We collected the presence of non-communicable chronic diseases (NCDs) of the participants by the question of “Have you been diagnosed with hypertension/diabetes/chronic obstructive pulmonary disease/cancer/periodontitis/other chronic diseases by a doctor?”. The response to presence of NCDs included “yes” and “no”. Activities of daily living were assessed using the Activities of Daily Living Scale (ADLs). ADLs is a scale with 14 terms that refers to peoples’ daily self-care activities which is used as a measurement of people’s functional status, especially in people with injuries or disabilities or older adults [29]. Total score of ADL ranges from 14 to 56, and the higher total score corresponds to worse ADL status [30]. The total score of ADL can be divided into 2 levels: normal (14 scores), and disabled (>14 scores).

Loneliness was assessed with the University of California Los Angeles Loneliness Scale (ULS) short-form (ULS-8), which consists of eight questions and each question is scored from 1 to 4 [31]. The total score of ULS-8 ranged from 8 to 32, with a high score indicating greater loneliness.

Psychological distress was measured by the Kessler Psychological Distress Scale (K10), which is an effective tool to evaluate people’s psychological status designed by Kessler, Andrews and so on [32]. It is a scale with 10-items regarding people’s level of anxiety and depression symptoms. The reliability and validity of K10 in Chinese-language version was demonstrated to be good [33]. The total score of K10 ranges from 10 to 50, and the higher score means more severe psychological distress.

Life Satisfaction was measured by the Satisfaction with Life Scale (SWLS) which was developed by Diener [34]. The scale includes 5 items and participants were asked to answer each item on a 7-point Likert scale (from 1 = strongly disagree to 7 = strongly agree). The total score ranges from 5 to 35, and the higher total score refers to higher life satisfaction.

### Statistical analysis

All statistical analyses were performed using SPSS 24.0. First, we presented descriptive statistics of the sample by gender, using the t test and Chi-square test to examine the difference between males and females. Then, binary logistic regression with one unadjusted model and one adjusted model were employed to examine the influencing factors associated with suicidal ideation among elderly. Finally, two separate binary logistic regression models were performed to determine the factors associated with suicidal ideation among males and females, respectively. All reported CIs were calculated at the 95% level. Statistical significance was set at the 5% level.

## Results

### Sample description

Table 1 shows the characteristics of the sample and the prevalence of suicidal ideation among rural elderly. Among the total 5514 rural elderly, 2366 were males (42.9%) and 3148 were females (57.1%). More than half of the participants aged 60–69 years (53.8%). Most of them were currently married (80.8%). As for education level, 2164 (39.2%) were illiterate, 2277 (41.3%) finished primary education, and 1073 (19.5%) finished junior school or above education. Regarding economic status, 856 (15.5%) reported good self-rated economic and 310 (5.6%) had debts. In terms of physical health status, 2636 (47.8%) reported moderate or bad self-rated health, 3796 (68.8%) suffered from NCDs, and 1418 (25.7%) was disabled. Besides, 775 (14.1%) lived alone, 296 (1.1%) had moderate or bad relationship with family, 2681 (48.6%) had stress of daily life, and 595 (10.8%) experienced setbacks or unfortunate events in the past two years. The elderly’s ULS-8 mean score was 11.09 ± 4.59, K10 mean score was 15.54 ± 6.87 and SWLS mean score was 30.55 ± 5.13.

**Table 1:** Characteristics of rural elderly by gender

Characteristics	Total *N* (%)	Male *N* (%)	Female N (%)	*χ*^2^/*t*	*P* value
Observations	5514	2366 (42.9)	3148 (57.1)		
Suicidal ideation				28.535	< 0.001
No	5089 (92.3)	2236 (94.5)	2853 (90.6)		
Yes	425 (7.7)	130 (5.5)	295 (9.4)		
Age				21.988	< 0.001
60~	2964 (53.8)	1195 (50.5)	1769 (56.2)		
70~	2063 (37.4)	968 (40.9)	1095 (34.8)		
80~	487 (8.8)	203 (8.6)	284 (9.0)		
Marital status				82.862	< 0.001
Married	4454 (80.8)	2043 (86.3)	2411 (76.6)		
Others	1060 (19.2)	323 (13.7)	737 (23.4)		
Education level				749.074	< 0.001
Illiterate	2164 (39.2)	477 (20.2)	1687 (53.6)		
Primary school	2277 (41.3)	1140 (48.2)	1137 (36.1)		
Junior school or above	1073 (19.5)	749 (31.7)	324 (10.3)		
Self-rated economic				16.279	< 0.001
Good	856 (15.5)	421 (17.8)	435 (13.8)		
Moderate or poor	4658 (84.5)	1945 (82.2)	2713 (86.2)		
Debts				3.564	0.059
No	5204 (94.4)	2217 (93.7)	2987 (94.9)		
Yes	310 (5.6)	149 (6.3)	161 (5.1)		
Self-rated health				19.989	< 0.001
Good	2878 (52.2)	1317 (55.7)	1561 (49.6)		
Moderate or bad	2636 (47.8)	1049 (44.3)	1587 (50.4)		
NCDs				52.919	< 0.001
No	1718 (31.2)	861 (36.4)	857 (27.2)		
Yes	3796 (68.8)	1505 (63.6)	2291 (72.8)		
ADL				28.962	< 0.001
Normal	4096 (74.3)	1844 (77.9)	2252 (71.5)		
Disabled	1418 (25.7)	522 (22.1)	896 (28.5)		
Living arrangement				43.806	< 0.001
Alone	775 (14.1)	248 (10.5)	527 (16.7)		
Others	4739 (85.9)	2118 (89.5)	2621 (83.3)		
Relationship with family				0.712	0.399
Good	5218 (94.6)	2232 (94.3)	2986 (94.9)		
Moderate or bad	296 (5.4)	134 (5.7)	162 (5.1)		
Stress of daily life				0.086	0.769
No	2833 (51.4)	1221 (51.6)	1612 (51.2)		
Yes	2681 (48.6)	1145 (48.4)	1536 (48.8)		
Negative life events				0.022	0.882
No	4919 (89.2)	2109 (89.1)	2810 (89.3)		
Yes	595 (10.8)	257 (10.9)	338 (10.7)		
ULS-8	11.09 ± 4.59	11.15 ± 4.59	11.05 ± 4.59	0.760	0.447
Psychological distress	15.54 ± 6.87	14.72 ± 6.58	16.15 ± 7.02	− 7.754	< 0.001
Life satisfaction	30.55 ± 5.13	30.59 ± 5.18	30.52 ± 5.10	0.497	0.619

The result showed that 425 (7.7%) elderly reported suicidal ideation within the past 12 months, including 130 males (5.5%) and 295 females (9.4%). There was statistically significant difference in suicidal ideation between rural males and females (*P* < 0.001). Besides, age (*P* < 0.001), marital status (*P* < 0.001), education level (*P* < 0.001), self-rated economic (*P* < 0.001), self-rated health (*P* < 0.001), NCDs (*P* < 0.001), ADL status (*P* < 0.001), living arrangement (*P* < 0.001) and psychological distress (*P* < 0.001) were also significantly different between males and females.

### Factors associated with suicidal ideation among rural male seniors

Table 2 shows the factors related to suicidal ideation among males. The results of adjusted binary logistic regression model revealed the following factors were statistically associated with suicidal ideation (*P* < 0.05), including education level, debts, stress of daily life, loneliness and psychological distress. Among males, those who were illiterate (adjusted OR = 1.98, *P* < 0.05) and finished primary education (adjusted OR = 1.89, *P* < 0.05), had debts (adjusted OR = 1.98, *P* < 0.05), had stress of daily life (adjusted OR = 1.70, *P* < 0.05), had greater feeling of loneliness (adjusted OR = 1.09, *P* < 0.001) and had severer psychological distress (adjusted OR = 1.09, *P* < 0.001) were positively significantly associated with suicidal ideation.

**Table 2:** Factors associated with the suicidal ideation among rural males

Characteristics	Suicidal ideation (N/%)		Unadjusted model	Adjusted model
	No	Yes	OR (95% CI)	OR (95% CI)
Variables	2236 (94.5)	130 (5.5)		
Age (reference: 60~)				–
70~	918 (94.8)	50 (5.2)	0.86 (0.59–1.25)	
80~	194 (95.6)	9 (4.4)	0.73 (0.36–1.49)	
Marital status (reference: married)				
Others	296 (91.6)	27 (8.4)	1.72 (1.11–2.67)*	1.18 (0.69–2.02)
Education level (reference: junior school or above)				
Illiterate	444 (93.1)	33 (6.9)	2.07 (1.22–3.50)**	1.98 (1.08–3.65)*
Primary school	1069 (93.8)	71 (6.2)	1.85 (1.17–2.92)**	1.89 (1.11–3.20)*
Self–rated economic (reference: good)				
Moderate or poor	1822 (93.7)	123 (6.3)	3.99 (1.85–8.62)***	1.35 (0.59–3.06)
Debts (reference: no)				
Yes	115 (77.2)	34 (22.8)	6.53 (4.23–10.08)***	1.98 (1.14–3.42)*
Self–rated health (reference: good)				
Bad	956 (91.1)	93 (8.9)	3.37 (2.28–4.97)***	1.29 (0.80–2.10)
NCDs (reference: no)				
Yes	1400 (93.0)	105 (7.0)	2.51 (1.61–3.91)***	1.63 (0.97–2.73)
ADL (reference: normal)				
Disabled	478 (91.6)	44 (8.4)	1.88 (1.29–2.74)**	0.64 (0.39–1.03)
Living arrangement (reference: others)				–
Alone	230 (92.7)	18 (7.3)	1.40 (0.84–2.35)	
Relationship with family (reference: good)				
Bad	106 (79.1)	28 (20.9)	5.52 (3.48–8.75)***	1.02 (0.55–1.88)
Stress of daily life (reference: no)				
Yes	1039 (90.7)	106 (9.3)	5.09 (3.24–7.99)***	1.70 (1.01–2.87)*
Negative life events (reference: no)				
Yes	216 (84.0)	41 (16.0)	4.31 (2.90–6.40)***	1.42 (0.88–2.31)
ULS-8	10.78 ± 4.11	17.52 ± 7.09	1.22 (1.19–1.26)***	1.09 (1.05–1.14)***
Psychological distress	14.12 ± 5.87	25.04 ± 9.15	1.17 (1.14–1.19)***	1.09 (1.06–1.13)***
Life satisfaction	30.88 ± 4.79	25.51 ± 8.19	0.88 (0.86–0.90)***	0.98 (0.95–1.01)

* *P* < 0.05; **P < 0.01; ***P < 0.001

### Factors associated with suicidal ideation among rural female seniors

The results of adjusted binary logistic regression model among females are represented in Table 3. The results showed that education level, debts, stress of daily life, negative life events, loneliness, psychological distress and life satisfaction were statistically associated with suicidal ideation (*P* < 0.05). For females, those who were illiterate (adjusted OR = 2.58 *P* < 0.01), had debts (adjusted OR = 1.96, *P* < 0.01), had stress of daily life (adjusted OR = 2.92, *P* < 0.001), experienced negative life events in past 2 years (adjusted OR = 2.42, *P* < 0.001), had greater feeling of loneliness (adjusted OR = 1.09, *P* < 0.001), had severer psychological distress (adjusted OR = 1.09, *P* < 0.001) and had lower life satisfaction (adjusted OR = 0.97, *P* < 0.05) and were more likely to have experienced suicidal ideation than their counterparts.

**Table 3:** Factors associated with the suicidal ideation among rural females

Characteristics	Suicidal ideation (*N*/%)		Unadjusted model	Adjusted model
	No	Yes	OR (95% CI)	OR (95% CI)
Variables	2853 (90.6)	295 (9.4)		
Age (reference: 60~)				–
70~	1001 (91.4)	94 (8.6)	0.85 (0.65–1.10)	
80~	260 (91.5)	24 (8.5)	0.83 (0.53–1.30)	
Marital status (reference: married)				–
Others	655 (88.9)	82 (11.1)	1.29 (0.99–1.69)	
Education level (reference: junior school or above)				
Illiterate	1499 (88.9)	188 (11.1)	2.01 (1.24–3.28)**	2.58 (1.47–4.52)**
Primary school	1049 (92.3)	88 (7.7)	1.35 (0.81–2.25)	1.70 (0.95–3.05)
Self-rated economic (reference: good)				
Moderate or poor	2437 (89.8)	276 (10.2)	2.48 (1.54–3.99)***	0.99 (0.57–1.71)
Debts (reference: no)				
Yes	107 (66.5)	54 (33.5)	5.75 (4.04–8.18)***	1.96 (1.25–3.07)**
Self-rated health (reference: good)				
Bad	1381 (87.0)	206 (13.0)	2.47 (1.90–3.20)***	0.97 (0.70–1.35)
NCDs (reference: no)				
Yes	2043 (89.2)	248 (10.8)	2.09 (1.52–2.89)***	1.13 (0.77–1.66)
ADL (reference: normal)				
Disabled	766 (85.5)	130 (14.5)	2.15 (1.68–2.74)***	1.18 (0.87–1.60)
Living arrangement (reference: others)				–
Alone	467 (88.6)	60 (11.4)	1.30 (0.97–1.76)	
Relationship with family (reference: good)				
Bad	116 (71.6)	46 (28.4)	4.36 (3.03–6.28)***	0.78 (0.48–1.26)
Stress of daily life (reference: no)				
Yes	1284 (83.6)	252 (16.4)	7.16 (5.14–9.98)***	2.92 (2.02–4.22)***
Negative life events (reference: no)				
Yes	229 (67.8)	109 (32.2)	6.72 (5.11–8.82)***	2.42 (1.75–3.37)***
ULS-8	10.51 ± 3.93	16.32 ± 6.68	1.21 (1.19–1.24)***	1.09 (1.06–1.12)***
Psychological distress	15.24 ± 6.13	24.93 ± 8.76	1.17 (1.15–1.18)***	1.09 (1.07–1.12)***
Life satisfaction	30.94 ± 4.53	26.43 ± 7.78	0.88 (0.86–0.90)***	0.97 (0.95–1.00)*

* *P* < 0.05; **P < 0.01; ***P < 0.001

## Discussion

Our study showed that the self-reported 12-month prevalence of suicidal ideation was 7.7% among 5514 rural elderly in Shandong Province, China. According to previous researches, the prevalence of suicidal ideation in rural seniors aged 60 and over in China ranges from 7.1 to 21.5%. A study performed in Hubei Province showed the prevalence of 12-month suicidal ideation among rural seniors was 7.1% [35]. A study conducted in Hunan Province, the reported suicidal ideation among rural elderly was 14.5% [20]. Another study from Hunan Province showed that the prevalence of suicidal ideation among rural elderly was 21.5% [36]. Different levels of socioeconomic development, research backgrounds, sample sizes and applied measures might explain the variations of the prevalence of suicidal ideation between our study and those studies mentioned above.

We also found that there was a significant gender difference towards suicidal ideation among elderly and the 12-month prevalence of suicidal ideation in females was significantly higher than that in males, which was in accordance with the findings from previous studies [10, 36, 37]. There might be three explanations for this finding. The first explanation is that in many Chinese rural families, females are dependent on males and they have to obey their husbands, their status is low in families. This lack of individual social identity and family status may increase the risk for psychological problems such as stress or depression which could then lead to the occurrence of suicidal ideation among these females. Second, rural females not only undertake more responsibility of taking care of the family, but also suffer more pressure from society and family. In such situation, females may be overwhelmed by negative emotion, feeling of hopelessness, lack of social support, problem solving, and coping skills in dealing with their stressful life event. Third, females are generally more vulnerable than males and have poor psychological tolerance when confronted with negative life events such as loss of a family or be diagnosed with cancer. Previous study illustrated that women more often reported a strong negative impact on psychological well-being when they encountered adverse life events [50]. So, once something bad happened to them, females are more likely to have suicidal ideation than males.

In this study, we revealed the different influencing factors of suicidal ideation between rural older males and females, few of which were mentioned in previous studies. And several influence factors were found to be associated with suicidal ideation in both males and females, such as education level, debts, stress of daily life, loneliness and psychological distress. Lower education level was an independent risk factor for suicidal ideation; this finding was consistent with the conclusions from some studies conducted in China, French and Korea [12, 38, 39]. Generally speaking, adults in poor economic circumstances were more likely to exhibit suicidal ideation than those in good economic circumstances. Many studies had demonstrated that population in debt were associated with higher incidence of suicidal ideation [40, 41], and this was also true for Chinese elderly. Another study revealed that financial loss rather than low income remained a significant correlated of suicidal ideation after controlling for depression [42]. Our findings were also in line with the results of previous researches, which suggested that loneliness was significantly correlated with suicidal ideation [43–45]. In addition, we also found that psychological distress was significantly associated with suicidal ideation among elderly, which was in agreement with previous studies [46, 47]. Elderly with higher score of psychological distress were more likely to have suicidal ideation, which implied that seniors began to consider that life was not worth living when they were in bad mental health. Furthermore, stress was also an important factor associated with suicidal ideation. Previous study suggested that suicide could function as an escape mechanism for avoiding stressful situations [48]. Similarly, our study showed that stress of daily life was significantly correlated with suicidal ideation in both males and females. These findings indicated that more attention should be paid to develop interventions targeting the at-risk subgroups to prevent suicidal ideation among the elderly in China.

However, two factors correlated with suicidal ideation were found to be different by gender. Among the females, negative life event was significantly associated with suicidal ideation, which was not found in males. People who encountered negative life events often experience psychological problems which contribute to suicidal ideation. The difference between males and females could be explained by the fact that females were more vulnerable when they experienced negative life events compared with males. And the role of female as a socially disadvantaged group made them more susceptible to crisis and negative emotions in setbacks, which made them more likely to have suicidal ideation. Another factor that was found to differ across gender was life satisfaction. Previous study showed that life satisfaction was inversely correlated with suicidal ideation among college students and this correlation tended to be stronger among the seniors than younger people [49]. In our study, females with lower life satisfaction had more likelihood to occur suicidal ideation, which was not found in males. One possible explanation might be that females suffered more pressure in taking care of family and dealing with household chores, so they had a stronger desire to live a good life. In our study, overall females reported lower life satisfaction than males, the imbalance of high life stress and low satisfaction with life might heighten the risk of suicidal ideation among these females. These findings implied that prevention by gender should be considered for preventing suicidal ideation and we should pay more attention to female elderly.

There are some limitations in this study. Firstly, the cross-sectional nature of our survey precludes us from assessing cause and effect to our results. Secondly, our data were based on self-reported measures such as self-rated economic status, relationship with family, stress of daily life, negative life event and NCDs; false or inaccurate response from participants may have led to recall bias or reporting bias. And measurement errors might exist because most variables were measured with single question. All of these may have influenced the validity of the results of our study. Thirdly, our main dependent variable was estimated via a single question instead of a standardized instrument, which could not fully reflect elderly’s diverse perspectives regarding suicidal ideation.

## Conclusions

Our study demonstrated that there was a significant gender difference towards suicidal ideation among rural elderly in Shandong, China. The common risk factors for suicidal ideation were education level, debts, stress of daily life, loneliness and psychological distress in males and females. We also found that negative life events and life satisfaction were significantly correlated with suicidal ideation in females but not in males. Thus, gender difference should be considered when medical practitioners and public health workers seek to prevent and manage suicidal ideation among rural elderly; more attention should be paid to females who had encountered negative life event or had lower life satisfaction.

## Data Availability

The datasets used in the current study are not publicly available due to the confidential policy, but are available from the corresponding author on reasonable request.

## Abbreviation

NCDs: non-communicable chronic diseases
ADL: Activity of Daily Living
ULS-8: University of California Los Angeles Loneliness Scale (ULS) short-form
OR: odds ratio
CI: confidence interval
